# Effects of COVID-19 on Physical Activity and Its Relationship With Mental Health in a US Community Sample: Cross-sectional, Convenience Sampling–based Online Survey

**DOI:** 10.2196/32387

**Published:** 2022-04-04

**Authors:** Wei Zhang, Dominick Velez

**Affiliations:** 1 Department of Psychology New Jersey City University Jersey City, NJ United States

**Keywords:** physical activity, COVID-19, mental health, depression, anxiety, United States, survey, cross-sectional, distress, risk

## Abstract

**Background:**

COVID-19 restrictions may make it difficult for people to engage in the recommended amounts of physical activity (PA).

**Objective:**

The influence of the COVID-19 pandemic on PA, as well as the links between PA and mental health, was investigated in this study.

**Methods:**

Participants were recruited using convenience sampling and responded to an online survey between April 15 and July 1, 2021, with ages ranging from 18 to 24 years (n=156, 40.9% of the sample) to ≥55 years (n=28, 7.4% of the sample). To assess general psychological distress, depression, anxiety, and pandemic anxiety, a battery of mental health assessments was used. The International Physical Activity Questionnaire - Short Form was used to collect PA data from participants, who were then classified as inactive, minimally active, or highly active. Participants also indicated the locations where they performed PA before and during COVID-19.

**Results:**

A sample of 381 individuals was included in this research. The logistic regression analysis results were interpreted as odds ratios (ORs), where an OR higher than 1 indicated a greater chance of an event occurring and an OR less than 1 implied a lower likelihood of an event occurring. Logistic regression results revealed that inactive individuals were more likely to develop psychological distress (OR 2.17, 95% CI 1.27-3.69, *P*=.004), depression (OR 3.81, 95% CI 1.92-7.57, *P*<.001), and anxiety (OR 1.86, 95% CI 0.99-3.47, *P*=.05) as compared to highly active individuals. Furthermore, when compared to highly active people, those who were only minimally active had a higher risk of depression (OR 2.14, 95% CI 1.05-4.33, *P*=.04). Wilcoxon signed-rank tests revealed that COVID-19 has a greater impact on reducing the chances of less active individuals engaging in PA outside and in public spaces. Highly active people's physical exercise locations had changed less, and their exercise frequency at home increased.

**Conclusions:**

Programmatic and policy interventions geared particularly toward enhancing PA among those less active may be a helpful strategy for addressing the worldwide pandemic’s mental health crisis.

## Introduction

### Background

The current COVID-19 pandemic has swept the world since the first confirmed case in Wuhan, China. COVID-19 is characterized by rapid transmission via droplets or close contact between humans. In the United States, over 33 million COVID-19 cases were reported as of July 1, 2021. Due to a lack of suitable treatments and vaccinations during the early stages of the pandemic, most countries adopted World Health Organization (WHO)-recommended protocols. Individuals worldwide were advised to stay at home and avoid contact with anyone who was not a close family member. Several studies have been published in recent months on the effects of COVID-19-related restrictions on psychological well-being, physical activity (PA), and general life satisfaction [1-9]. Although these self-quarantine methods were critical in reducing the spread of COVID-19, they may have limited people's capacity to engage in sufficient amounts of PA to preserve health and avoid illness.

During the pandemic, people worldwide were urged to stay at home and avoid contact with others. Businesses, organizations, and institutions, for example, encouraged their workers to work from home to ensure their own safety. People who worked from home had fewer opportunities to interact with coworkers and participate in fewer PAs, such as walking between meeting locations [10]. Students were no longer allowed to participate in school-based PA, such as physical education classes or walking to and from transportation, as they moved to online learning. The majority of team sports, league training, and games were canceled. Due to lockdowns and other restrictions, people found it impossible to access gyms, parks, and other places where they might exercise. Although prepandemic PA levels were already insufficient for many, pandemic management efforts are likely to have had the unintended consequence of further lowering PA. Early investigations did, in fact, show a substantial decrease in PA levels since the pandemic began [11].

The relationship between the COVID-19 restrictions and PA may be particularly significant, given that frequent and considerable physical exercise is essential for health and well-being in general [12]. Exercise has a main effect in treating depression [13,14]. Physical inactivity, in contrast, has both acute and long-term negative psychological consequences, as well as negative effects on people's metabolic, vascular, and immune systems across a wide range of age groups, races, genders, health conditions, and body shapes [15]. To maintain psychological and physical well-being during the pandemic, WHO recommended that people engage in physical exercise, particularly while in self-quarantine.

According to a study performed in the United States during the early stages of the COVID-19 pandemic in April 2020, participants reporting decreased PA experienced higher depression, loneliness, and stress [16]. Similarly, research conducted in April 2020 on adults in Italy and Australia revealed that participants reporting decreased PA following the pandemic were more likely to have negative psychological health and well-being [6,17]. Research conducted on Canadian participants from April to May 2020 revealed that those who were more physically active had better mental health and those who became more active or who had more PAs in the outdoors had less anxiety [18]. A survey study conducted between 2015 and 2020 discovered that during the pandemic in 2020, there was a significant decline in PA and mental health among college students, while PA did not appear to protect against deterioration in mental health, with participants drawn from a large northeastern US university and predominately females and non-Hispanic Whites [1]. In a rapid systematic review of COVID-19 research on physical exercise and depression and anxiety, the authors indicated there were methodological weaknesses in some of the studies, such as the use of unvalidated instruments and ﻿failure to provide standardized statistics [5]. Furthermore, since most of previous research was performed shortly after the pandemic outbreak [3], a new study of PA and its relationship with psychological health may be needed to generate further findings after the effect of a year-long pandemic restriction.

### Aims of This Study

In sum, there have been few up-to-date studies on the impact of pandemic restrictions on physical exercise and their connections to mental health. This research investigated whether physical exercise is linked to a decrease in anxiety and depression. In addition, we investigated whether there were any changes in the locations of participants’ PA before the COVID-19 outbreak and during the pandemic.

## Methods

### Participants and Procedure

Data were collected via an online, anonymous survey via Qualtrics from April 15 to July 1, 2021. The study used convenience sampling to recruit individuals aged 18 years and above from a public university campus community located in the metropolitan region of New Jersey, United States. The research was publicized to the university community, including students, faculty, and staff, through email, flyers, and campus announcements. The completed questionnaire was submitted by 381 respondents. The questionnaire included questions about demographics, lifestyle, and socioeconomic position, as well as key variables examined, such as mental health and PAs. The data provided here were centered on factors associated with changes in physical and mental well-being. Participants would enter the draw for a $50 gift card by providing their email addresses. As an added precaution, a range of security mechanisms in Qualtrics and a human check were implemented in the survey for removing potential bots and preventing multiple submissions.

### Ethics Approval

Before taking the survey, participants provided their electronic informed consent. Participants had the right to agree or refuse to participate in the research and withdraw at any time. The research was approved by the university's institutional review board (IRB).

### Measurements

#### Mental Health Problems (Psychological Distress, Depression, and Anxiety)

A broad and well-validated 4-item Patient Health Questionnaire (PHQ-4) was used to measure psychological distress [19,20]. The PHQ-4 includes 2 items that assess depressive symptoms (“feeling down, depressed or hopeless,” “little interest or pleasure in doing things”) and 2 items that assess anxiety (“feeling nervous, anxious, or on edge,” “not being able to stop or control worrying”). The questionnaire begins with the broad question, “Over the last 2 weeks, how often have you been bothered by the following problems?” Participants rate the number of times these problems have bothered them on a 4-point scale of 0 (*not at all*), 1 (*several days*), 2 (*more than half the days*), or 3 (*nearly every day*). The total score for the PHQ-4 ranges from 0 to 12, with higher scores indicating more symptoms of psychological distress. Following established protocols, the total score was transformed into 4 categories, which indicated various psychological distress levels: *none-to-minimal* (≤2), *mild* (3-5), *moderate* (6-8), and *severe* (9-12).

The PHQ-4 can be divided into 2 ultrabrief screening scales [21,22]: the 2-item Generalized Anxiety Disorder (GAD-2) scale for screening anxiety disorders and the PHQ-2 for screening depression disorders [23,24]. PHQ-2 and GAD-2 subscales have values ranging from 0 to 6, with higher scores suggesting more symptoms of depression and anxiety. A subscale score of 3 suggests a cut-off point between the normal range and probable clinical depression or anxiety disorder [20]. Using a cut-off value of 3, the PHQ-2 and GAD-2 scales were converted into binary variables, indicating a greater risk of depression and anxiety.

The PHQ-4 has been evaluated for construct validity in general [20,21,25], and it has been shown to correspond with suitable self-report measures and known demographic risk factors for depression and anxiety. In this research, the PHQ-4 had a Cronbach α of .88, while the GAD-2 and PHQ-2 subscales had a Cronbach α of .88 and .83, respectively. The reliabilities were comparable to those of other research that used the same scale in a similar context.

#### Pandemic Anxiety

A survey item assessing the prevalence of symptoms of physical anxiety linked to the COVID-19 outbreak was used to assess pandemic anxiety [26,27]. The survey question asked participants how often they had physical anxiety symptoms over a certain week, reflecting their COVID-19 experience: “In the past 7 days, have you had physical reactions, such as sweating, trouble breathing, nausea, or a pounding heart, when thinking about your experience with the COVID-19 pandemic?” Participants chose from the following list of options: *rarely or none of the time* (less than 1 day), *some or a little of the time* (1-2 days), *occasionally or a moderate amount of time* (3-4 days), or *most or all of the time* (5-7 days). This question has been modified from the Impact of Event Scale used by the American Psychiatric Association to capture reported physical discomfort following traumatic events. The criteria used are defined in the *Diagnostic and Statistical Manual of Mental Disorders* as symptoms of posttraumatic stress disorder [28]. Based on previous research, a binary measure was created by keeping the lowest variable level, *rarely or none of the time* (less than 1 day), as 0 and the 3 highest variable levels as 1 [27].

#### Physical Activity

The International Physical Activity Questionnaire - Short Form (IPAQ-SF) was used in the assessment of the levels of individuals' PA and had good reliability, as measured [29,30]. This questionnaire asks about PA done in the previous 7 days and details on the frequency and duration spent on 3 distinct levels of PA: vigorous-intensity activity (eg, aerobics), moderate-intensity activity (eg, carrying light load, bicycling at a regular pace), and walking activities. According to the official IPAQ scoring procedure, the participants of the study were classified into 3 different categories of PA (*highly active*, *minimally active*, and *inactive*), with the IPAQ analytic algorithms considering both the total PA volume and the number of days/sessions. Individuals who were highly active surpassed the minimal public health PA guidelines and accumulated enough exercise to maintain a healthy lifestyle. Individuals who were minimally active got the minimum amount of exercise recommended for adults in current public health guidelines but not enough to be highly active. Individuals that were inactive were engaged at the lowest level of PA, meaning they did not fulfil the minimal active requirements.

#### Physical Activity Locations

Participants were asked to report the frequency of the types of locations they used for PAs before the pandemic and during the COVID-19 outbreak to determine whether the pandemic impacted how individuals engaged in physical exercise. The following options were provided: parks/trails, botanical gardens, recreational sports/intramural facilities, neighborhoods, home-based activities (eg, workouts, housekeeping, fitness video games), fitness facilities (eg, gyms), time outdoors with animals, transportation to the workplace, and the workplace. Participants of the study indicated how often they used each of these locations for PA purposes over a 1-month period, on a scale of 0 (*never*), 1 (*rarely*), 2 (*sometimes*), and 3 (*frequently*), before and during the pandemic. This tool was developed by Michigan State University as part of the PhenxTookit for COVID-19 [31].

### Data Analyses

First, descriptive statistics on the main variables analyzed were reported, and gender differences (female vs male) were examined using chi-square and *t* tests for categorical data and continuous data, respectively. Differences between PA groups were investigated and are reported in Multimedia Appendix 1. Second, a series of ordinal and binary logistic regression analyses were conducted to examine the effects of PA (independent variable) on the probability of experiencing mental health issues (dependent variables), considering potential confounders (age, gender, education, marital status, smoking, and ethnicity) [32-35]. Dependent variables included psychological distress (categorical data), depression (binary data), anxiety (binary data), and pandemic anxiety (binary data). The results of the logistic regression analyses were presented as odds ratios (ORs) and their 95% CIs [36]. Third, we used Wilcoxon signed-rank tests to explore changes in the locations of participants’ PA between the pre-COVID-19 period and the COVID-19 period by different PA groups. Effect sizes were reported for the relevant statistics.

Participants were given the option of stopping the survey at any time or skipping a question if they did not feel comfortable answering it. Following the cleanup of missing and irregular data, there were a small number of participants lacking PA data (n=51, 13.4%) due to unanswered questions on the scale. For PA location data, missing data varied from 0.3% to 3.9%. The percentage of missing data for questions related to pandemic anxiety was 0.3%. Many sociodemographic variables (eg, gender, education, ethnicity, marital status) had missing data ranging from 0.3% to 1.6%. There was no difference in demographic distribution between individuals with missing data and those with full data. Missing data were preserved in the analysis sample and deleted pairwise. All analyses were accomplished with SPSS Statistics version 27.

## Results

### Sample Characteristics

Table 1 presents the sociodemographic and health characteristics of the sample. There were 287 (75.9%) females, 88 (23.3%) males, and 3 (0.8%) others. In addition, there were 156 (40.9%) participants in the 18-24-year age range, 164 (43.0%) in the 25-44-year age range, and 61 (16.0%) in the ≥45-year age range. Over half (n=249, 66.4%) of the participants were single or never married, and 207 (54.5%) of the individuals had a bachelor’s degree or above. About half of the participants (n=213, 56.3%) resided either in big cities or small cities. Almost three-quarters (n=278, 73.0%) of the sample reported being employed before COVID-19, while 67 (17.6%) reported having lost their job since the pandemic. The sample was racially diverse, with 41 (10.9%) Asians, 55 (14.6%) Black/Africans, 112 (29.7%) Caucasians, 124 (32.9%) Hispanic/Latino, and 35 (9.3%) others. Most of the participants did not smoke (n=338, 88.9%).

As assessed by the PHQ-4 (mean 4.4, SD 3.6), 134 (35.2%) of the individuals had none-to-mild, 123 (32.3%) had mild, 64 (16.8%) had moderate, and 60 (15.7%) had severe symptoms of psychological distress. In addition, 133 (34.9%) of the participants were likely to suffer from depression, whereas 145 (38.1%) of the individuals had probable anxiety. Nearly a quarter of the sample stated that they were concerned about a pandemic (n=89, 23.4%). Finally, according to the IPAQ-SF, 122 (37.0%) of 330 participants were categorized as inactive, 96 (29.1%) as minimally active, and 112 (33.9%) as highly active.

Table 2 depicts that no gender differences were observed for the majority of the variables examined, although females reported greater psychological distress and anxiety and were also less likely to be physically active (all *P*<.05).

**Table 1:** Sample sociodemographic characteristics.

Sociodemographic characteristics		Participants, n (%)
**Age (years; N=381)**		
	18-24	156 (40.9)
	25-44	164 (43.0)
	45-54	33 (8.7)
	55-64	17 (4.5)
	≥65	11 (2.9)
**Relationship status (N=375)**		
	Single, never married	249 (66.4)
	Married/domestic partnership	99 (26.4)
	Other	27 (7.2)
**Gender (N=378)**		
	Male	88 (23.3)
	Female	287 (75.9)
	Other	3 (0.8)
**Ethnicity (N=377)**		
	Asian	41 (10.9)
	Black/African	55 (14.6)
	Caucasians	112 (29.7)
	Hispanic/Latino	124 (32.9)
	Other	35 (9.3)
	Prefer not to say	10 (2.7)
**Education (N=380)**		
	High school/some high school	37 (9.7)
	Some college/associate degree	136 (35.8)
	Bachelor's degree	114 (30.0)
	Graduate/professional school	93 (24.5)
**Employment/student status (pre-COVID-19; N=381)^a^**		
	Employed (full-time, part-time, self-employed)	278 (73.0)
	Student (full-time, part-time)	158 (41.5)
**Employment/student status (post-COVID-19; N=381)^a^**		
	Still employed/studying but with decreased hours	56 (14.7)
	Still employed/studying but with increased hours	35 (9.2)
	Still employed/studying, moved to remote or hybrid work	105 (27.6)
	Lost job	67 (17.6)
	No change	124 (32.5)
**Household income change (post-COVID-19; N=377)**		
	My household income is more.	57 (15.1)
	My household income is less.	152 (40.3)
	My household income is about the same.	168 (44.6)
**Living location (N=378)**		
	Large city	114 (30.2)
	Small city	99 (26.2)
	Suburbs of a large city	59 (15.6)
	Town or village	80 (21.2)
	Rural area	12 (3.2)
	Don’t know	14 (3.7)
**Smoking (N=380)**		
	No	338 (88.9)
	Yes	42 (11.1)
**Psychological distress (N=381); mean (SD)=4.4 (3.58)**		
	None to mild	134 (35.2)
	Mild	123 (32.3)
	Moderate	64 (16.8)
	Severe	60 (15.7)
**Depression score≥3 (N=381); mean (SD)=2.09 (1.94)**		
	Yes	133 (34.9)
	No	248 (65.1)
**Anxiety score≥3 (N=381); mean (SD)=2.35 (1.96)**		
	Yes	145 (38.1)
	No	236 (61.9)
**Pandemic anxiety (N=380)**		
	Rarely or none of the time	291 (76.6)
	Some or a little of the time	64 (16.8)
	Occasionally or moderate amount of time	20 (5.3)
	Most of all the time	5 (1.3)
**PA^b^ (N=330)**		
	Inactive	122 (37.0)
	Minimally active	96 (29.1)
	Highly active	112 (33.9)

^a^Participants may choose multiple responses that apply to their situations.

^b^PA: physical activity.

**Table 2:** Sample characteristics split by sex.

Characteristics		Female, n/N (%)	Male, n/N (%)	Gender differences			
				*χ*^2^ (*df*)		*P* value^a^	
**Age (years)**					2.05 (2)		.36
	18-24	114/287 (39.7)	37/88 (42.0)	N/A^b^		N/A	
	25-44	122/287 (42.5)	41/88 (46.6)	N/A		N/A	
	≥45	51/287 (17.8)	10/88 (11.4)	N/A		N/A	
**Relationship status**					1.87 (2)		.39
	Single, never married	186/281 (66.2)	57/88 (64.8)	N/A		N/A	
	Married/domestic partnership	72/281 (25.6)	27/88 (30.7)	N/A		N/A	
	Others	23/281 (8.2)	4/88 (4.5)	N/A		N/A	
**Ethnicity**					11.02 (5)		.05
	Asian	28/284 (9.9)	12/88 (13.6)	N/A		N/A	
	Black/African	46/284 (16.2)	8/88 (9.1)	N/A		N/A	
	Caucasians	84/284 (29.6)	27/88 (30.7)	N/A		N/A	
	Hispanic/Latino	96/284 (33.8)	26/88 (29.5)	N/A		N/A	
	Others	26/284 (9.2)	9/88 (10.2)	N/A		N/A	
	Prefer not to say	4/284 (1.4)	6/88 (6.8)	N/A		N/A	
**Education**					1.97 (3)		.58
	High school/some high school	24/286 (8.4)	11/88 (12.5)	N/A		N/A	
	Some college/associate degree	99/286 (34.6)	33/88 (37.5)	N/A		N/A	
	Bachelor's degree	90/286 (31.5)	24/88 (27.3)	N/A		N/A	
	Graduate/professional school	73/286 (25.5)	20/88 (22.7)	N/A		N/A	
**Smoking**					0.28 (1)		.60
	No	256/286 (89.5)	77/88 (87.5)	N/A		N/A	
	Yes	30/286 (10.5)	11/88 (12.5)	N/A		N/A	
**Psychological distress: female mean (SD)=4.57 (3.65), male mean (SD)=3.761 (3.09), *t*_167.71_=2.05, *P*=.04**					7.12 (3)		.07
	None to minimal	101/287 (35.2)	32/88 (36.4)	N/A		N/A	
	Mild	85/287 (29.6)	37/88 (42.0)	N/A		N/A	
	Moderate	54/287 (18.8)	10/88 (11.4)	N/A		N/A	
	Severe	47/287 (16.4)	9/88 (10.2)	N/A		N/A	
**Depression: female mean (SD)=2.105 (1.99), male mean (SD)=1.921 (1.69), *t*_167.81_=0.86, *P*=.39**					0.06 (1)		.81
	Yes (score≥3)	97/287 (33.8)	31/88 (35.2)	N/A		N/A	
	No	190/287 (66.2)	57/88 (64.8)	N/A		N/A	
**Anxiety: female mean (SD)=2.46 (1.98), male mean (SD)=1.841 (1.69), *t*_167.43_=2.91, *P*=.004**					5.23 (1)		.02
	Yes (score≥3)	117/287 (40.8)	24/88 (27.3)	N/A		N/A	
	No	170/287 (59.2)	64/88 (72.7)	N/A		N/A	
**Pandemic anxiety**					0.64 (1)		.42
	Less than 1 day	223/286 (78.0)	65/88 (73.9)	N/A		N/A	
	At least 1 day	63/286 (22.0)	23/88 (26.1)	N/A		N/A	
**PA^c^**					27.10 (2)		<.001
	Inactive	106/253 (41.9)	16/74 (21.6)	N/A		N/A	
	Minimally active	79/253 (31.2)	14/74 (18.9)	N/A		N/A	
	Highly active	68/253 (26.9)	44/74 (59.5)	N/A		N/A	

^a^The *P* values represent chi-square/*t* tests of independence, indicating associations between sex and categorical variables. Categories were created for age (18-24, 25-44, and ≥45 years).

^b^N/A: not applicable.

^c^PA: physical activity.

### Logistic Regression Analyses

We used ordinal and binary logistic regressions to estimate the ORs (and 95% CIs) for the association of PA (independent variable; categorical) with various mental health outcomes, with the confounding factors of gender, age, race, educational level, marital status, and smoking considered (Tables 3-6). Psychological distress (categorical), depression (binary), anxiety (binary), and pandemic anxiety (binary) were dependent variables.

**Table 3:** ORs^a^ for logistic regression analyses for physical levels and mental health outcome (psychological distress).

	Characteristics	OR (95% CI)	*P* value
**Gender (reference “male,” n=74)**		1.75 (1.01-3.02)	.05
**Age (years; reference≥45, n=46)**			
	25-44	2.98 (1.37-6.49)	.006
	18-24	3.60 (1.39-9.32)	.008
**Ethnicity (reference “Caucasians,” n=99)**			
	Black/Africans	1.38 (0.67-2.83)	.39
	Asian	1.79 (0.85-3.77)	.13
	Hispanic/Latino	0.89 (0.51-1.56)	.68
	Others	1.18 (0.54-2.61)	.68
	Prefer not to say	4.58 (0.96-21.79)	.06
**Education (reference “graduate/professional school,” n=77)**			
	Bachelor's degree	1.53 (0.83-2.84)	.18
	Some college/associate degree	2.56 (1.28-5.14)	.008
	High school/some high school	4.37 (1.71-11.19)	.002
**Marriage status (reference “married/domestic partnership,” n=84)**			
	Single/never married	2.49 (1.35-4.59)	.003
	Others	1.99 (0.75-5.25)	.16
**Smoking (reference “no smoking,” n=280)**		4.35 (2.26-8.38)	<.001
**PA^b^ levels (reference “highly active,” n=111)**			
	Minimally active	1.52 (0.87-2.65)	.14
	Inactive	2.17 (1.27-3.69)	.004

^a^OR: odds ratio.

^b^PA: physical activity.

**Table 4:** ORs^a^ for logistic regression analyses for physical levels and mental health outcome (depression).

	Characteristics	OR (95% CI)	*P* value
**Gender (reference “male,” n=74)**		0.87 (0.44-1.71)	.69
**Age (years; reference≥45, n=46)**			
	25-44	4.50 (1.36-14.92)	.01
	18-24	5.23 (1.32-20.76)	.02
**Ethnicity (reference “Caucasians,” n=99)**			
	Black/Africans	0.93 (0.37-2.34)	.88
	Asian	0.77 (0.30-1.95)	.58
	Hispanic/Latino	0.46 (0.23-0.94)	.03
	Others	0.64 (0.23-1.76)	.39
	Prefer not to say	1.03 (0.15-7.24)	.98
**Education (reference “graduate/professional school,” n=77)**			
	Bachelor's degree	1.33 (0.59-3.02)	.50
	Some college/associate degree	2.88 (1.19-6.99)	.02
	High school/some high school	7.14 (2.19-23.25)	.001
**Marriage status (reference “married/domestic partnership,” n=84)**			
	Single/never married	1.67 (0.76-3.63)	.20
	Others	1.57 (0.42-5.90)	.50
**Smoking (reference “no smoking,” n=280)**		3.42 (1.56-7.51)	.002
**PA^b^ levels (reference “highly active,” n=111)**			
	Minimally active	2.14 (1.05-4.33)	.04
	Inactive	3.81 (1.92-7.57)	<.001

^a^OR: odds ratio.

^b^PA: physical activity.

**Table 5:** ORs^a^ for logistic regression analyses for physical levels and mental health outcome (anxiety).

	Characteristics	OR (95% CI)	*P* value
**Gender (reference “male,” n=74)**		1.91 (0.98-3.70)	.06
**Age (years; reference≥45, n=46)**			
	25-44	1.57 (0.60-4.12)	.36
	18-24	1.85 (0.59-5.81)	.29
**Ethnicity (reference “Caucasians,” n=99)**			
	Black/Africans	1.57 (0.68-3.65)	.29
	Asian	1.36 (0.56-3.30)	.50
	Hispanic/Latino	0.80 (0.41-1.57)	.52
	Others	0.99 (0.38-2.56)	.98
	Prefer not to say	1.53 (0.24-9.73)	.65
**Education (reference “graduate/professional school,” n=77)**			
	Bachelor's degree	1.76 (0.82-3.77)	.15
	Some college/associate degree	1.97 (0.84-4.66)	.12
	High school/some high school	2.17 (0.71-6.62)	.18
**Marriage status (reference “married/domestic partnership,” n=84)**			
	Single/never married	3.08 (1.45-6.55)	.003
	Others	1.25 (0.35-4.55)	.73
**Smoking (reference “no smoking,” n=280)**		2.16 (1.02-4.56)	.04
**PA^b^ levels (reference “highly active,” n=111)**			
	Minimally active	1.60 (0.83-3.08)	.16
	Inactive	1.86 (0.99-3.47)	.05

^a^OR: odds ratio.

^b^PA: physical activity.

**Table 6:** ORs^a^ for logistic regression analyses for physical levels and mental health outcome (pandemic anxiety).

	Characteristics	OR (95% CI)	*P* value
**Gender (reference “male,” n=74)**		0.77 (0.38-1.56)	.47
**Age (years; reference≥45, n=46)**			
	25-44	4.23 (1.10-16.22)	.04
	18-24	2.90 (0.61-13.69)	.18
**Ethnicity (reference “Caucasians,” n=99)**			
	Black/Africans	2.29 (0.84-6.20)	.10
	Asian	1.11 (0.39-3.15)	.85
	Hispanic/Latino	2.01 (0.92-4.35)	.08
	Others	0.84 (0.24-2.90)	.78
	Prefer not to say	2.78 (0.41-18.99)	.30
**Education (reference “graduate/professional school,” n=77)**			
	Bachelor's degree	0.98 (0.41-2.38)	.97
	Some college/associate degree	1.39 (0.54-3.58)	.49
	High school/some high school	1.72 (0.51-5.79)	.38
**Marriage status (reference “married/domestic partnership,” n=84)**			
	Single/never married	0.74 (0.33-1.64)	.45
	Others	0	.99
**Smoking (reference “no smoking,” n=280)**		1.84 (0.84-4.03)	.13
**PA^b^ levels (reference “highly active,” n=111)**			
	Minimally active	1.41 (0.65-3.06)	.39
	Inactive	1.72 (0.84-3.54)	.14

^a^OR: odds ratio.

^b^PA: physical activity.

#### Psychological Distress

According to ordinal logistic regression, inactive people were 2.17 times more likely than highly active people to have higher levels of psychological distress (OR 2.17, 95% CI 1.27-3.69, *P*=.004).

Regarding confounding factors, individuals aged 18-24 years (OR 3.60, 95% CI 1.39-9.32, *P=*.008) and 25-44 years (OR 2.98, 95% CI 1.37-6.49, *P*=.006) were more likely to experience psychological distress than those aged ≥45 years. Females were 1.75 times more likely to experience greater psychological distress than males (OR 1.75, 95% CI 1.01-3.02, *P*=.05). Those who had just finished high school or some high school (OR 4.37, 95% CI 1.71-11.19, *P*=.002) and some college or associate degree (OR 2.56, 95% CI 1.28-5.14, *P*=.008) were more likely to experience psychological distress than those who had completed a graduate or professional school program. Single individuals had a higher risk of distress (OR 2.49, 95% CI 1.35-4.59, *P*=.003) than married people or those in a domestic partnership. Participants who smoked had increased odds of psychological distress (OR 4.35, 95% CI 2.257-8.381, *P*<.001).

#### Depression

Logistic regression results revealed a significant relationship between PA levels and depression (*P*<.001). Minimally active individuals were 2.14 times more likely to suffer from depression than highly active people (OR 2.14, 95% CI 1.05-4.33, *P*=.04). The odds of having depression were 3.81 times greater among inactive individuals than among highly active individuals (OR 3.81, 95% CI 1.92-7.57, *P*<.001).

Furthermore, individuals aged 18-24 years (OR 5.23, 95% CI 1.32-20.76, *P*=.02) and 25-44 years (OR 4.5, 95% CI 1.36-14.92, *P*=.01) had higher odds of depression than those aged ≥45 years. Completing just high school or some high school (OR 7.14, 95% CI 2.19-23.25, *P*=.001) and some college/associate degree (OR 2.88, 95% CI 1.19-6.99, *P*=.02) was connected to greater odds of having depression. Individuals who smoked were 3.422 times more likely to suffer from depression than those who did not (OR 3.422, 95% CI 1.56-7.51, *P*=.002).

#### Anxiety

We found no significant relationship between PA levels and anxiety disorder in binary logistic regression, though the odds of anxiety were marginally higher among inactive participants than among highly active participants (OR 1.86, 95% CI 0.99-3.47, *P*=.05).

Gender marginally predicted the odds of anxiety (*P*=.06), where females had 1.91 more chances of having anxiety than males (OR 1.91. 95% CI 0.984-3.702). Single individuals had greater odds of having anxiety than married people or those in a domestic partnership (OR 3.08, 95% CI 1.45-6.55, *P*=.003). Smoking increased the odds of anxiety 2.16-fold (OR 2.16, 95% CI 1.023-4.555, *P*=.04).

#### Pandemic Anxiety

We found no significant connection between PA levels and pandemic anxiety in logistic regression analysis or between major demographic factors and pandemic anxiety; however, those aged 25-44 years (OR 4.23, 95% CI 1.10-16.22, *P*=.04) were more likely to suffer from pandemic anxiety than those aged ≥45 years.

### Changes in Locations of PA Following COVID-19

Wilcoxon signed-rank tests were conducted to examine the changes in the frequency of usage of various PA-performing locations before and during the COVID-19 outbreak and how they might vary for inactive, minimally active, and highly active people (Table 7). Inactive individuals during COVID-19 were less likely to engage in physical exercise at parks/trails (Z=–4.01, *P*<.001), botanical gardens (Z=–2.02, *P*=.04), recreational sports or intramural facilities (Z=–3.04, *P*=.002), neighborhood sidewalks and parks (Z=–3.78, *P*<.001), and fitness facilities (Z=–4.35, *P*<.001); during commuting to work (Z=–4.696, *P*<.001); and at their workplace (Z=–3.19, *P.*<001). The results for the minimally active individuals were largely the same as those for the inactive individuals such that the probability of performing PA at parks/trails (Z=–1.90, *P*=.06), botanical gardens (Z=–2.52, *P*=.01), recreational sports and intramural facilities (Z=–2.52, *P*=.01), and fitness facilities (Z=–4.29, *P*<.001) and during transport to the workplace (Z=–3.53, *P*<.001) were ranked less frequently during COVID-19. However, minimally active individuals spent a comparable amount of time performing PA in their neighborhood (*P=*.91) and at work (*P*=.52). For highly active people, although the frequency of performing PA in botanical gardens (Z=–2.545, *P*=.01) and fitness centers (Z=–4.71, *P*<.001), during transit to work (Z=–3.82, *P*<.001), and at the workplace (Z=–2.33, *P*=.02) decreased, the frequency of performing PA in parks/trails (*P*=.17), recreational sports/intramural facilities (*P*=.16), and neighborhoods (*P*=.45) did not change significantly. Furthermore, only highly active individuals increased their PA at home (eg, workouts, housekeeping, yard work, gardening, exercise, video games) during the pandemic (Z=2.93, *P*=.003). Finally, the likelihood of engaging in PA outdoors with animals did not change, and this was the same for all 3 PA groups (all *P*>.30).

**Table 7:** Wilcoxon signed-rank tests: comparing locations^a^ of PA^b^ before and during COVID-19 (split by 3 PA groups).

Ranks			Parks/trails		Botanical gardens		Recreational sports/intramural facilities		Neighborhoods		Home-based activity		Fitness facilities		Time outdoors with animals		Transportation to workplace		Workplace
**Inactive**																			
	Negative ranks^c^	40		15		17		40		29		30		12		39		19	
	Positive ranks^d^	13		4		4		15		22		5		13		7		5	
	Ties^e^	64		96		93		61		65		82		92		69		91	
	Z value^f^	–4.01		–2.02		–3.04		–3.78		–1.59		–4.35		0.18		–4.70		–3.19	
	*P* value^f^	<.001		.04		.002		<.001		.12		<.001		.86		<.001		.001	
**Minimally active**																			
	Negative ranks	28		21		24		20		21		34		17		26		14	
	Positive ranks	15		6		8		16		19		5		11		7		9	
	Ties	52		66		63		59		54		56		67		61		72	
	Z value	–1.90		–2.52		–2.52		–0.11		–0.10		–4.29		–1.00		–3.53		–0.65	
	*P* value	.06		.01		.01		.91		.92		<.001		.32		<.001		.52	
**Highly active**																			
	Negative ranks	18		25		25		15		15		44		11		34		18	
	Positive ranks	34		7		15		18		31		9		14		7		7	
	Ties	59		77		68		77		65		57		86		68		83	
	Z value	1.37		–2.55		–1.39		0.76		2.93		–4.71		0.32		–3.82		–2.33	
	*P* value	.17		.01		.16		.45		.003		<.001		.75		<.001		.02	

^a^Frequency scores of PA locations were rated as 0=never, 1=rarely, 2=sometimes, and 3=frequently.

^b^PA: physical activity.

^c^Negative ranks: during COVID-19 < before COVID-19.

^d^Positive ranks: during COVID-19 > before COVID-19.

^e^Ties: post-COVID-19 score=pre-COVID-19 score.

^f^Z and *P* values represent Wilcoxon signed-rank tests indicating differences.

## Discussion

### Principal Findings

In this study, we examined the impact of pandemic restrictions on physical exercise and its connections to mental health in a community sample of adults. Our results showed that those who engaged in greater PA during the COVID-19 pandemic had less psychological distress, depression, and anxiety than those who engaged in less PA. Furthermore, COVID-19 was found to make it hard for people to keep up with their PA habits, especially for people who were less active in using outdoor and public PA facilities.

According to the data, approximately 133 (34.9%) and 145 (38.1%) of the sample scored above the cut-offs for depression and anxiety, respectively; about two-thirds of individuals reported mild-to-severe psychological distress. The logistic regression illustrated that not engaging in PA during COVID-19 was related to about 1.86-3.81 times’ higher risks of psychological discomfort, depression, and anxiety disorders. The highest degree of PA (exceeding the minimum public health PA recommendations and accumulating enough activity for a healthy lifestyle) but not moderate PA (fulfilling minimal PA recommendation) appeared to be associated with lower psychological risk during a pandemic [9]. These results were essentially consistent with the majority of prior studies on the relationship between PA and COVID-19, showing that PA would protect people's mental health in general and lessen their risk of depression and anxiety [5,6,9,17,18,37]. The outcomes of this study might help people live healthy, resilient lives both during and after the worldwide pandemic.

When examining the different types of mental health problems, there was some evidence from logic regression analyses that PA might affect depression more than anxiety. Participants who were inactive had marginally higher odds of anxiety than those who were highly active, while those who were inactive or minimally active had higher odds of depression than those who were highly active. One explanation might be a stark temporal differentiation between the natures of depression and anxiety. For example, participants reported depression-triggering events taking place in their past, relating to loss and failures in achieving goals, whereas anxiety-triggering events were related to fears about the future [38]. In the unique case of the COVID-19 pandemic, 1 of the most salient factors individuals were challenged with was the inherent inability to predict how long restrictions and legitimate health risks would prevail, putting enormous doubt on life in the future. Although an inability to control current circumstances might be combatted by decreasing cortisol levels and achieving personal fitness goals through PA, positively affecting depression levels, remedies for anxiety about the prevalence of the pandemic and an inability to project what is to come cannot be manufactured. Our findings were consistent with a recent study performed during COVID-19 on a Chinese sample between February and March 2020, which found that the link between PA and depression was more robust than the association between PA and anxiety [39]. Research conducted in a sample of older adults over the age of 50 years who lived in North America in April 2020 found that PA was not a significant predictor of anxiety symptoms after controlling for age, sex, and education, while PA was a significant predictor for depressive symptoms [7]. A meta-analysis of the relationship between PA and depression and anxiety in nonclinical adult populations found that PA decreased depression with a medium effect size and anxiety with a small effect size [40], independent of the pandemic. A quick systematic review conducted during the pandemic found a similar result: the relationship between PA and depression was more consistent than the relationship between PA and anxiety [5]. Further analysis is needed to find the other additional factors that are linked to anxiety in the global pandemic.

Our results revealed that the amount of time people spent on PA at various locations varied before and after the start of COVID-19. In general, reduced PA seemed to be linked to a lack of sporting opportunities. COVID-19 had a greater impact on reducing the use of PA resources (such as parks/trails, recreational sports, neighborhoods) by people who were classified as less active or minimally active. Highly active individuals might have been affected less and have adapted to doing their PA in the comfort of their homes. Individuals who participated vigorously in regular physical exercise might have acquired PA-related health literacy, which enabled them to use it as a typical coping technique for negative emotions, resulting in the fast adoption of new sports routines. Health care providers and the government may urge inactive individuals to include PAs in their everyday lives to reduce the rise in mental health issues during the current pandemic. Given the uncertainties surrounding the return to normalcy, encouraging at-home activities seems to be a viable solution. Virtual reality (VR) technologies (eg, Oculus Quest) and other home-based commercial video games (eg, Nintendo Switch) have been shown to improve PA in settings that are more pleasant and entertaining, which is especially appropriate when movement in the outdoor world is restricted [41-43].

Demographic factors associated with mental health risks included age, gender, smoking, education, and marital status, although their effects might vary depending on the kind of mental health problems studied. Females were more prone to stress, anxiety, and depression than males according to previous research [44,45]. We found that females had higher psychological distress and anxiety odds than males during the pandemic. Previous research revealed that younger people were more likely to have worse mental health than older people, and risk factors, such as loneliness and financial hardship, were more likely to impact younger people than others during the COVID-19 pandemic [45-47]. Our study found that those over 45 years had the lowest psychological distress and depression ratings. Smoking is frequently associated with poor mental health, regardless of the pandemic [48]. According to our results, individuals who smoked had higher levels of psychological distress and higher levels of depression and anxiety. In contrast to marriage, being single or divorced has been associated with poorer mental health [49]. Our study reported that those in marriage or domestic relationships experienced lower stress and anxiety during the pandemic. Our findings also revealed that higher levels of education were critical determinants related to greater positive well-being [50]. Furthermore, our supplementary analysis results indicated gender, racial, and age differences in PA, consistent with previous research [51]. To sum up, many of these health-related demographic variables had been identified in previous research prior to the pandemic. These findings suggest that sociodemographic risk factors would be linked to the risk of physical and mental health during the pandemic.

We have achieved considerable progress in studying the effects of physical exercise on psychological health during the pandemic. Wolf et al [5] noted methodological issues, such as using unvalidated measures and the inability to offer standardized coefficients, in their most recent review paper published in 2021 evaluating publications on associations between PA and depression and anxiety during COVID-19. Wolf et al [5] also stated that studies included in their review used heterogeneous statistical approaches (eg, multiple linear regression, logistic regression) and study designs (eg, cross-sectional, longitudinal design), which would have yielded a more sophisticated overall effect estimate of the relationships between PA and depression and anxiety. Based on our effect size measures, our study found similar effects of physical exercises on reducing mental health problems as prior studies conducted in a comparable context [9,52]. Furthermore, our study included data from later in the pandemic, enabling future researchers to get a more complete picture of the effect of PA on pandemic mental health.

### Limitations

The study's limitations included the use of a cross-sectional research methodology. As a result, the causal nature of these relationships was unknown. PA was not linked to the pandemic anxiety and could have been attributed to lower COVID-19-related physical anxiety symptoms in individuals. Of 380 participants, 291 (76.6%) reported that they had few or non-pandemic-related concerns. Participants might have been expected to be increasingly adapted and prepared as the pandemic progresses. Furthermore, although we followed the previous study of measuring pandemic-related anxiety [27], a single question measuring anxiety related to the pandemic in the past week may not possibly sum up all the characteristics of pandemic-related stress, and additional research is necessary. Another limitation worth mentioning was the sampling method [53]. The study used a convenience sample approach by recruiting university community members. Such samples could attract volunteers who were already engaged and interested in the issue and had internet access. Previous research suggested that people who were currently suffering from or had a serious mental illness might be less likely to participate online than those who were not [53-55]. The study also included a larger female sample, although a more balanced sample was anticipated. As we have seen, the sampling method is an important issue in research, and we will continue with caution when extrapolating our findings to a different population setting.

Because of the COVID-19-related restrictions on human subject research during the pandemic, we were unable to examine the mechanisms through which physical exercise could help people's mental health via collecting neurobiological data in the study. This warrants further investigation by other researchers. The biological pathway through which PA affects mental health is likely via the hypothalamic-pituitary-adrenal (HPA) axis regulation [56,57]. Physical fitness and physical exercise, for example, were shown to be associated with reduced cortisol release when subjects were subjected to psychosocial stressors [58]. Additionally, recent research indicated that exercise's positive benefits on the brain were associated with possible underlying biological processes, including the gut-brain, muscle-brain, and liver-brain axes [59]. Future studies may also explore the effect of COVID-19 on PA in countries with more substantial or fewer restrictions to see whether there are differences. Research in the future may focus on establishing and evaluating interventions that help people maintain regular exercise as part of their daily lives throughout and after the pandemic.

### Conclusion

In summary, the results from this study showed that people who did more PA during the COVID-19 pandemic had less psychological distress, depression, and anxiety than people who engaged in less PA. Additionally, it was found that COVID-19 interrupted people's opportunities, particularly those less active in using outdoor and public PA facilities to keep PA habits. Without a doubt, the pandemic's influence, particularly the long-term effects, cannot be predicted at the moment. Promoting and encouraging PA behavior is a cost-effective way of creating a necessary distinction between those who have a basic mental health need and those who are mentally well. This strategy is particularly beneficial in communities with inadequate health resources [60].

## Appendix

Multimedia Appendix 1 Supplementary analysis.

## Abbreviations

GAD-2: 2-item Generalized Anxiety Disorder
IPAQ-SF: International Physical Activity Questionnaire - Short Form
OR: odds ratio
PA: physical activity
PHQ-4: 4-item Patient Health Questionnaire
