# Supplementary material for: Effects of COVID-19 on Physical Activity and Its Relationship With Mental Health in a US Community Sample: Cross-sectional, Convenience Sampling–based Online Survey
Source: JMIR Form Res. 2022 Apr 4;6(4):e32387. doi: 10.2196/32387 (PMC8982649; doi:10.2196/32387)

**Supplement Results**

There was a significant association between PA group distribution and age (*P=.*04). From the corresponding graph (Figure 1S.A), it appears the 25-44 group has a higher proportion of highly active people, while the 18-25 and ≥45 age groups have a higher proportion of physically inactive and minimally active individuals. PA groups and gender association were found to be significant (*P<.*001), with more females in the inactive and minimally active groups and men in the highly active groups (Figure 1S.B). PA groups and ethnicity association were found to be significant (*P=*.04) with relatively more physically active individuals in the Caucasian ethnic category (Figure 1S.C). The distribution of PA groups was unrelated to smoking, education, and marital status variables.

The distribution of PA groups differed for different degrees of psychological distress (*P=.*02). Relatively more highly active individuals were in the none-to-minimal and mild stress groups, while more inactive and minimally active people were in the moderate and severe groups (Figure 1S.D). The distribution of PA groups differed between those with depression and those without (*P=*.003). There were more highly active people in the no depression than in the depression group (Figure 1S.E). The PA group difference was significant for anxiety (*P=*.050), with no anxiety group having more highly active individuals, while the anxiety group had more people who were less active (Figure 1S.F).

**Table S1. Comparisons by PA levels**

|  |  | **PA Groups** | | | | | |
| --- | --- | --- | --- | --- | --- | --- | --- |
|  |  | Inactive | | Minimally active | | High active | |
| **Gender, n, (%)** | | χ^2^ = 27.10, df = 2, *P<*.001 | | | | | |
|  | Male | 16 | (13.1%) | 14 | 15.1%) | 44 | 39.3% |
|  | Female | 106 | (86.9%) | 79 | 84.9% | 68 | 60.7% |
| **Age, n, (%)** | | χ^2^ = 10.07, df = 4, *P=.*04 | | | | | |
|  | 18-24 | 55 | (45.1%) | 45 | 46.9% | 37 | 33% |
|  | 25-44 | 46 | (37.7%) | 35 | 36.5% | 62 | 55.4% |
|  | ≥45 | 21 | (17.2%) | 16 | 16.7% | 13 | 11.6% |
| **Ethnicity, n, (%)** | | χ^2^ = 19.29, df = 10, *P=.*04 | | | | | |
|  | Caucasians | 21 | (17.4%) | 39 | 415% | 41 | 36.6% |
|  | Black/Africans | 19 | (15.7%) | 11 | 11.7% | 14 | 12.5% |
|  | Asian | 16 | (13.2%) | 10 | 10.6% | 11 | 9.8% |
|  | Hispanic/Latino | 50 | (41.3%) | 27 | 28.7% | 31 | 27.7% |
|  | Others | 13 | (10.7%) | 6 | 6.4% | 12 | 10.7% |
|  | Prefer Not Say | 2 | (1.7%) | 1 | 1.1% | 3 | 2.7% |
| **Education, n, (%)** | | χ^2^ **=** 1.8, df = 6, *P=.*94 | | | | | |
|  | High school/some high school | 15 | (12.4%) | 9 | 9.4% | 9 | 8% |
|  | Some college/Associate degree | 43 | (35.5%) | 34 | 35.4% | 37 | 33% |
|  | Bachelor's degree | 36 | (29.8%) | 30 | 31.3% | 37 | 33% |
|  | Graduate/Professional School | 27 | (22.3%) | 23 | 24% | 29 | 25.9% |
| **Relationship status, n, (%)** | | χ^2^ = 1.15, df = 4, *P=.*89 | | | | | |
|  | Single, never married | 79 | (65.8%) | 62 | 65.3% | 77 | 69.4% |
|  | Married/domestic partnership | 31 | (25.8%) | 27 | 28.4% | 28 | 25.2% |
|  | Others | 10 | (8.3%) | 6 | 6.3% | 6 | 5.4% |
| **Smoking, n, (%)** | | χ^2^ **=** 0.78, df = 2, *P=.*68 | | | | | |
|  | No | 110 | (90.2%) | 83 | 87.4% | 97 | 86.6% |
|  | Yes | 12 | (9.8%) | 12 | 12.6% | 15 | 13.4% |
| **Psychological distress, n, (%)** | | χ^2^ = 14.57, df = 6, *P=.*02 | | | | | |
|  | None-to-minimal | 34 | (27.9%) | 36 | 37.5% | 43 | 38.4% |
|  | Mild | 36 | (29.5%) | 23 | 24% | 44 | 39.3% |
|  | Moderate | 27 | (22.1%) | 17 | 17.7% | 14 | 12.5% |
|  | Severe | 25 | (20.5%) | 20 | 20.8% | 11 | 9.8% |
| **Depression, n, (%)** | | χ^2^ = 11.35, df = 2, *P=.*003 | | | | | |
|  | No, PHQ-2 ≤ 2 | 66 | (54.1%) | 58 | 60.4% | 84 | 75% |
|  | Yes, PHQ-2 ≥ 3 | 56 | (45.9%) | 38 | 39.6% | 28 | 25% |
| **Anxiety, n, (%)** | | χ^2^ = 5.98, df = 2, *P=.*050 | | | | | |
|  | No, GAD-2 ≤ 2 | 68 | (55.7%) | 56 | 58.3% | 79 | 70.5% |
|  | Yes, GAD-2 ≥ 3 | 54 | (44.3%) | 40 | 41.7% | 33 | 29.5% |
| **Pandemic Anxiety, n, (%)** | | χ^2^ **=** 2.07, df = 2, *P=.*36 | | | | | |
|  | Rarely | 92 | (75.4%) | 75 | 78.1% | 93 | 83% |
|  | Not Rarely | 30 | (24.6%) | 21 | 21.9% | 19 | 17% |

Note: The *P* values represent chi-square tests of independence indicating associations between PA groups and variables.

Figure 1S. The distribution of physical activity groups by age, gender, ethnicity, and mental health variables.

Figure 1S. A.


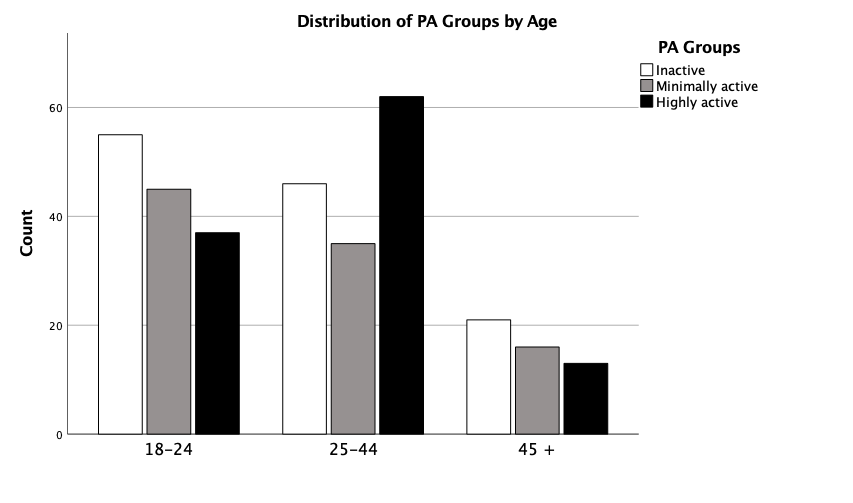


Figure 1S. B.


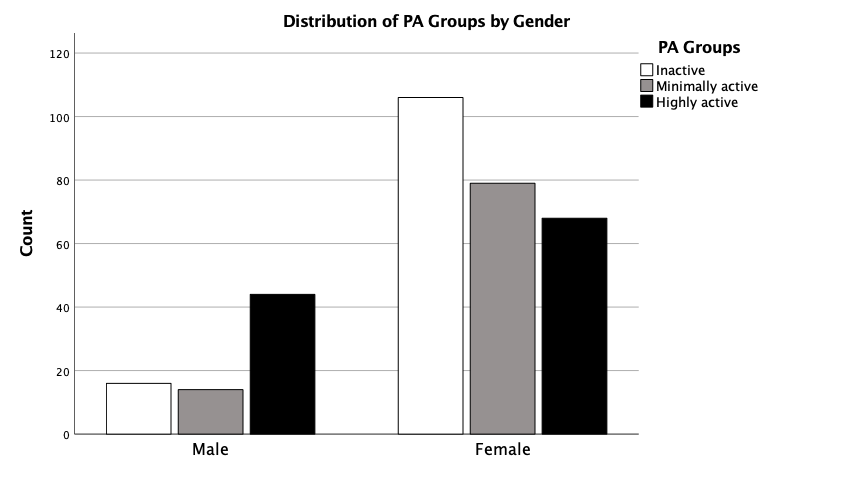


Figure 1S. C.


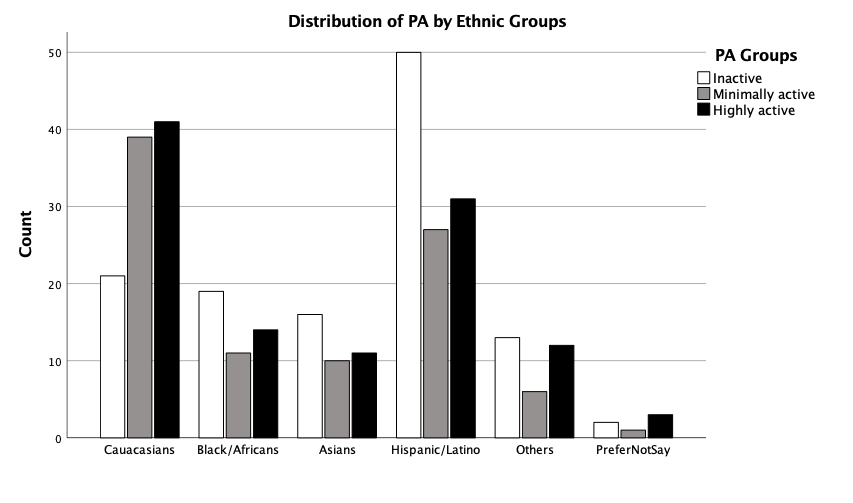


Figure 1S. D.


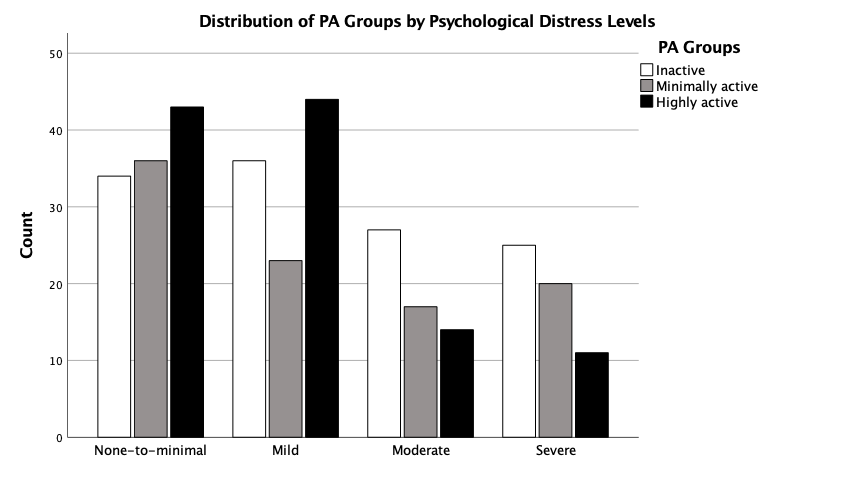


Figure 1S. E.


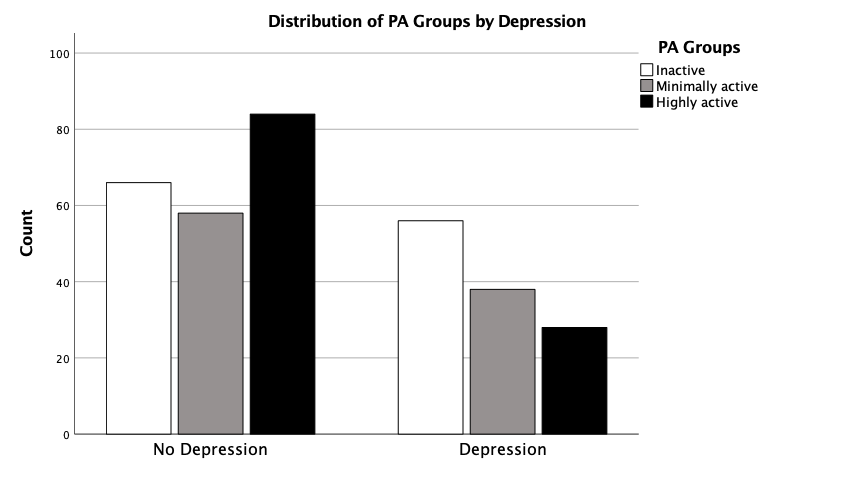


Figure 1S. F.


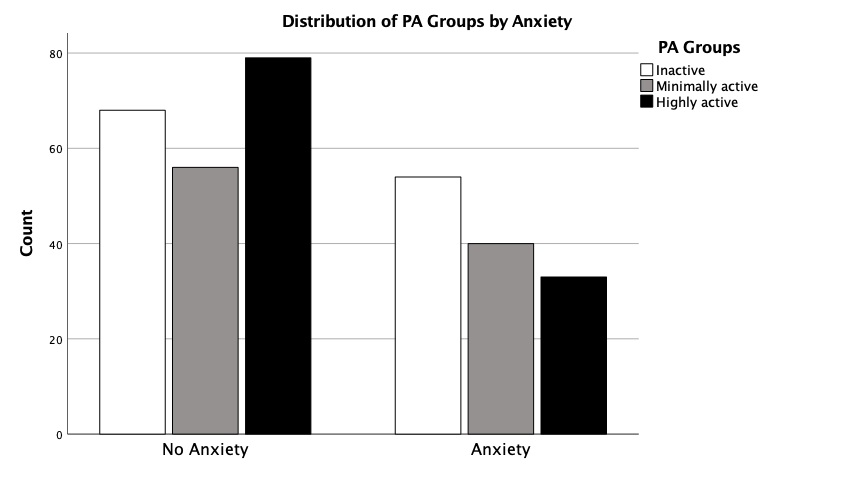

Supplement: Multimedia Appendix 1 [file formative_v6i4e32387_app1.docx]
